# Supplementary material for: An excitatory lateral hypothalamic circuit orchestrating pain behaviors in mice
Source: eLife. 2021 May 27;10:e66446. doi: 10.7554/eLife.66446 (PMC8159376; doi:10.7554/eLife.66446)
Supplement: Transparent reporting form [file elife-66446-transrepform.docx]

***eLife’s* transparent reporting form**

We encourage authors to provide detailed information *within their submission* to facilitate the interpretation and replication of experiments. Authors can upload supporting documentation to indicate the use of appropriate reporting guidelines for health-related research (see [EQUATOR Network](http://www.equator-network.org/%20)), life science research (see the [BioSharing Information Resource](https://biosharing.org/)), or the [ARRIVE guidelines](http://www.plosbiology.org/article/info:doi/10.1371/journal.pbio.1000412) for reporting work involving animal research. Where applicable, authors should refer to any relevant reporting standards documents in this form.

If you have any questions, please consult our Journal Policies and/or contact us: [editorial@elifesciences.org](mailto:editorial@elifesciences.org).

**Sample-size estimation**

- You should state whether an appropriate sample size was computed when the study was being designed
- You should state the statistical method of sample size computation and any required assumptions
- If no explicit power analysis was used, you should describe how you decided what sample (replicate) size (number) to use

Please outline where this information can be found within the submission (e.g., sections or figure legends), or explain why this information doesn’t apply to your submission:

- Sample size estimates for functional imaging studies are described in the *Methods* section in the first paragraph under subheading *In Vivo Functional Imaging.*
- Sample size estimates for behavioral studies are described in the *Methods* section in the first paragraph under subheading *Behavioral Experiments.*
- Sample size estimates for the recombinant rabies virus tracing study are described in the *Methods* section in the first paragraph under subheading *Recombinant Rabies Virus Tracing.*
- Final group sizes for imaging and behavioural studies are reported in the Figure Legends. The group size and cell count numbers from the rabies virus tracing are reported in the results section and within Figure 5.

**Replicates**

- You should report how often each experiment was performed
- You should include a definition of biological versus technical replication
- The data obtained should be provided and sufficient information should be provided to indicate the number of independent biological and/or technical replicates
- If you encountered any outliers, you should describe how these were handled
- Criteria for exclusion/inclusion of data should be clearly stated
- High-throughput sequence data should be uploaded before submission, with a private link for reviewers provided (these are available from both GEO and ArrayExpress)

Please outline where this information can be found within the submission (e.g., sections or figure legends), or explain why this information doesn’t apply to your submission:

- This study included many different behavioral tests, the frequency of each is reported within the respective methods section or described in the narrative of the results section.
- The definition of biological versus technical replication is included in the *Methods* section under subheading *Animals*.
- Biological replicates (e.g., group sizes) are indicated in the Results section and Figure Legends. Technical replications (e.g., von Frey filament stimulations) are described for each assay in the Methods section.
- No outliers were encountered in this study.
- Exclusion criteria are described in the *Methods* section under subheadings *Behavioral Experiments* and *Recombinant Rabies Virus Tracing.*

**Statistical reporting**

- Statistical analysis methods should be described and justified
- Raw data should be presented in figures whenever informative to do so (typically when N per group is less than 10)
- For each experiment, you should identify the statistical tests used, exact values of N, definitions of center, methods of multiple test correction, and dispersion and precision measures (e.g., mean, median, SD, SEM, confidence intervals; and, for the major substantive results, a measure of effect size (e.g., Pearson's r, Cohen's d)
- Report exact p-values wherever possible alongside the summary statistics and 95% confidence intervals. These should be reported for all key questions and not only when the p-value is less than 0.05.

Please outline where this information can be found within the submission (e.g., sections or figure legends), or explain why this information doesn’t apply to your submission:

(For large datasets, or papers with a very large number of statistical tests, you may upload a single table file with tests, Ns, etc., with reference to sections in the manuscript.)

- Statistical analyses are detailed in the *Methods* section and are specifically noted for each comparison in the Figure Legends.
- Individual data points are shown for calcium transient rate during the formalin test, for all place preference figures, for paw licking data, and for supplemental figures showing nociceptive sensitization. Individual data points were omitted from some of the other bar graphs (e.g., Figure 2) as the group size surpassed N = 10 and the data was sometimes discrete (e.g., zones cleared) rather than continuous. Individual data points were omitted from line graphs with optogenetics since the group averages (ChR2 vs control) in several of these graphs overlapped (e.g., Figure 6b-d) and individual data points would muddle their appearance; data points were left off of the non-overlapping graphs (e.g., Figure 3b) for consistency. For the chemogenetic experiments in Figure 7, several tests’ worth of data is already included in each panel; indicating individual data points would greatly obscure the graphs.
- The statistical tests, exact values of N, definition of center, multiple comparisons correction, and measures of dispersion are all addressed in the Figure Legends (for the cell counts, also in the Results section). Cohen’s *d* effect sizes are reported alongside select comparisons in the Figure Legends.
- Exact p-values are reported down to *p* = 0.0001, which is the threshold of our statistical software, any values lower are annotated as *p* < 0.0001.

**Group allocation**

- Indicate how samples were allocated into experimental groups (in the case of clinical studies, please specify allocation to treatment method); if randomization was used, please also state if restricted randomization was applied
- Indicate if masking was used during group allocation, data collection and/or data analysis

Please outline where this information can be found within the submission (e.g., sections or figure legends), or explain why this information doesn’t apply to your submission:

- Mice were randomly assigned to experimental groups to obtain groups that were gender-matched and age-matched. This information is stated in the *Methods* section under subheading *Behavioral Experiments*.
- Blinding was implemented during data collection of the nesting behavior and of data analysis for formalin paw licking behavior; this is stated in the *Methods* section for each of these assays.

**Additional data files (“source data”)**

- We encourage you to upload relevant additional data files, such as numerical data that are represented as a graph in a figure, or as a summary table
- Where provided, these should be in the most useful format, and they can be uploaded as “Source data” files linked to a main figure or table
- Include model definition files including the full list of parameters used
- Include code used for data analysis (e.g., R, MatLab)
- Avoid stating that data files are “available upon request”

Please indicate the figures or tables for which source data files have been provided:

- Source data for Figure 1 has been provided as numerical time series data in spreadsheets.
